# Comment on Meneghini et al. The Impact of Nutritional Therapy in the Management of Overweight/Obese PCOS Patient Candidates for IVF. *Nutrients* 2023, *15*, 4444

**DOI:** 10.3390/nu16030417

**Published:** 2024-01-31

**Authors:** Nenad Cetkovic, Giuseppe Guido Maria Scarlata, Ludovico Abenavoli

**Affiliations:** 1 Department of Obstetrics and Gynecology, Clinical Center of Vojvodina, Faculty of Medicine, University of Novi Sad, 21000 Novi Sad, Serbia; nenad.cetkovic@mf.uns.ac.rs; 2 Department of Health Sciences, University “Magna Græcia”, 88100 Catanzaro, Italy; giuseppeguidomaria.scarlata@unicz.it

We read with great interest the recent article by Meneghini et al. on the assessment of the effects of different alimentary regimens, included Mediterranean diet (MD), on polycystic ovary syndrome (PCOS) patients prior to in vitro fertilization cycles [1]. Lifestyle changes, focused on healthy diet, have been reported as the first-line approach in the management of PCOS, with the primary aim to normalize metabolic features and to restore the reproductive profile [2]. Data from the literature suggest that MD may be useful in the prevention and in the management of gonadal disorders [3]. These beneficial effects could be related to antioxidant properties of MD and its high rate of fruits, vegetables, whole grains, and heart-healthy fats [4]. Additionally, MD eliminates processed meats, saturated fats, and refined sugar, which worsen systemic inflammatory status [4]. Recently, scientific interest in antioxidant-rich foods has increased, as epidemiology has shown an inverse relationship between the adoption of a diet rich in nutraceuticals and the onset of metabolic disorders [5]. MD is a complete alimentary model characterized by the richness of natural compounds, with different biological activities such as phenols, flavonoids, and sterols. In healthy subjects, several trials have shown the efficacy of healthy diet in gene expression and metabolic pathways, suggesting that the inflammatory status reflects the antioxidant properties of foods [5]. In this context, metabolic diseases, including PCOS, show altered pathways of oxidative stress [6]. For this reason, it is necessary to identify therapeutic strategies that contemplate the use of personalized diet rich in antioxidants as MD.

Polyphenols are heterogeneous plant-derived molecules that include several hydro-soluble antioxidants commonly used in the maintenance of good health and the prevention of different metabolic conditions [7]. Currently, there is an increasing volume of evidence on the effects of food rich in polyphenols on PCOS and its accompanying metabolic features such as insulin resistance, lipid profile impairment, inflammation, and oxidative stress [8]. In recent years, our group have confirmed the role of MD in body fat mass reduction and the improvement of lipid profile and insulin resistance parameters [9]. Furthermore, the synergic effect of MD and a healthy lifestyle has been evaluated in patients with liver steatosis, with a significant reduction in or regression of hepatic fat accumulation and improvement of liver enzyme levels and anthropometric parameters [10]. Supported by our data and according to Meneghini et al., we can conclude that MD associated with healthy lifestyle changes is a useful approach to normalize metabolic features in patients with metabolic syndrome or PCOS. We support the use of MD as a treatment and as a preventive measure to improve the clinical management of these subjects. However, more large randomized clinical trials are needed to confirm these data.
